# The Treatment of Club Foot

**Published:** 1902-06-28

**Authors:** 


### THE TREATMENT OF CLUB FOOT.

Professor Ogston[1] has devised and carried out a highly ingenious method of treating cases of club foot which have either not been treated in the first year or two of life or in whom the treatment at that age has not been satisfactory. The method is applicable up to about six years of age, and perhaps a year or two later. It depends upon the fact that the tarsal bones, which oppose the chief obstacles to the rectification of club foot, do not complete their ossification for many years, while in children suffering from club foot their ossification is an even slower process. In healthy children they consist of a rounded kernel of bone enclosed in a thick layer of cartilage whose ossification will eventually complete their external configuration. Now, if the whole or a considerable part of a tarsal bone be resected for club foot, that bone or portion of bone is lost; but if an incision be made through the soft parts at some suitable spot, and deepened so as to divide the enclosing layer of unossified cartilage, the bony kernel may be partially or completely removed by a sharp spoon, and the bone will be restored by ossification from the shell of cartilage which remains. When a tarsal bone has been thus deprived of its osseous centre it is soft and plastic, and very moderate pressure suffices to mould it into any required shape, so that it ceases to offer any notable obstacle to the rectification of the deformity produced by the club foot. It is possible moreover by gliding the wound in the soft parts to deal with several bones in the same way through one external incision. Professor Ogston commences the operation by the usual disinfection of the skin, the application of an Esmarch's tourniquet, and the performance of tenotomy on the tendo Achillis. Then he makes an incision which runs in a gentle curve, beginning in front of the external malleolus and extending forwards, with its convexity towards the sole, until it terminates over the calcaneo-cuboid joint on its dorsal aspect, and through this incision the bony kernel of the astragalus is scooped out, and if necessary those of the os calcis and of the cuboid are removed also. But in all cases the cartilaginous shell must be left intact, and the joints must not be opened. The foot is then moulded into the desired shape and fixed in plaster of Paris. The results as shown by photographs and skiagraphs appear to be admirable. No joint is opened, the ligaments and other important structures are not interfered with, and perfect motion in all the articulatus is obtained.

[1] Brit. Med. Jour., June 21.

